# “THE MANTLE” bundle for minimizing cerebral hypoxia in severe traumatic brain injury

**DOI:** 10.1186/s13054-022-04242-3

**Published:** 2023-01-12

**Authors:** Daniel Agustin Godoy, Francisco Murillo-Cabezas, Jose Ignacio Suarez, Rafael Badenes, Paolo Pelosi, Chiara Robba

**Affiliations:** 1 Departamento Medicina Critica. Unidad de Cuidados Neurointensivos, Sanatorio Pasteur, Catamarca, Argentina; 2 Departamento de Medicina, Universidad de Sevilla, Sevilla, Spain; 3 Departments of Anesthesiology and Critical Care Medicine, Neurology, and Neurosurgery, The Johns Hopkins University School of Medicine, Baltimore, USA; 4 Anesthesiology and Surgical-Trauma Intensive Care, University Clinic Hospital, Valencia, Spain; 5 Department of Surgery, University of Valencia, Valencia, Spain; 6 INCLIVA Research Medical Institute, Valencia, Spain; 7 Anesthesia and Intensive Care, San Martino Policlinico Hospital, IRCCS for Oncology and Neurosciences, Genoa, Italy; 8 Department of Surgical Sciences and Integrated Diagnostics, University of Genoa, Genoa, Italy

**Keywords:** Brain oxygenation, Cerebral oxygenation monitoring, Brain hypoxia, Cerebral ischemia, Traumatic brain injury

## Abstract

To ensure neuronal survival after severe traumatic brain injury, oxygen supply is essential. Cerebral tissue oxygenation represents the balance between oxygen supply and consumption, largely reflecting the adequacy of cerebral perfusion. Multiple physiological parameters determine the oxygen delivered to the brain, including blood pressure, hemoglobin level, systemic oxygenation, microcirculation and many factors are involved in the delivery of oxygen to its final recipient, through the respiratory chain. Brain tissue hypoxia occurs when the supply of oxygen is not adequate or when for some reasons it cannot be used at the cellular level. The causes of hypoxia are variable and can be analyzed pathophysiologically following “the oxygen route.” The current trend is precision medicine, individualized and therapeutically directed to the pathophysiology of specific brain damage; however, this requires the availability of multimodal monitoring. For this purpose, we developed the acronym “THE MANTLE,” a bundle of therapeutical interventions, which covers and protects the brain, optimizing the components of the oxygen transport system from ambient air to the mitochondria.

## Introduction

**Fig. 1:**
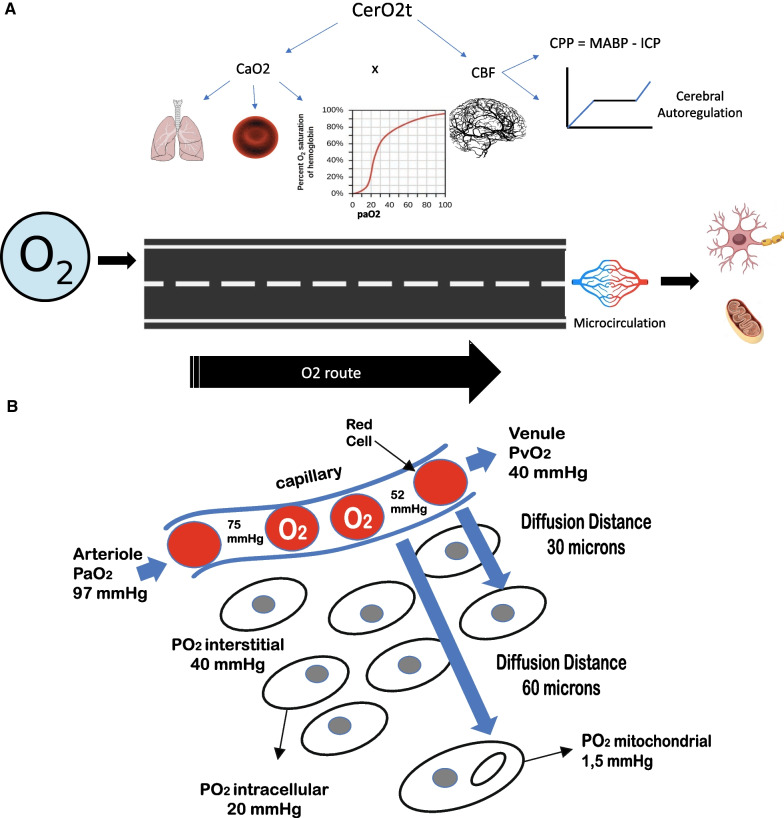
**A** Oxygen, O_2_ route. From atmospheric air or the gaseous mixture supplied by mechanical ventilation, O_2_ travels following concentration gradients. Cerebral O_2_ transport (CerO_2_t) depends on the product of CBF and arterial O_2_ content (CaO_2_), determined by the following equation: CaO_2_ = (Hgb × 1.34 x SaO_2_) + (PaO_2_ × 0.003), where: Hgb: concentration in gr/dl; 1.34: number of ml transported by each gram of Hgb; SaO_2_: arterial O_2_ saturation; PaO_2_: arterial pressure of O_2_. The affinity of oxygen for Hgb is expressed by analyzing the Hgb-oxygen saturation curve. The CBF is mainly determined by the cerebral perfusion pressure (CPP) and the radius of the cerebral resistance vessels (autoregulation curve). **B **O_2_ diffusion at cellular level. If the physiological variables interact harmoniously, oxygen reaches the microcirculation at 98 mmHg, then diffuses into the cell through the interstitial space (PO_2_i = 20–40 mmHg). Inside the cell, the O_2_ pressure is 1.5 mmHg. The distance that the 0_2_ must travel varies between 20 and 60 microns

**Fig. 2:**
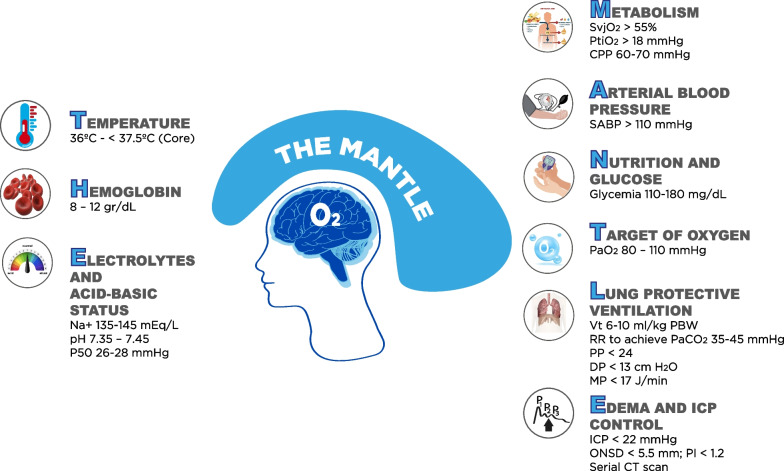
The MANTLE mnemonics. Jugular venous saturation of oxygen, SvjO_2_; brain tissue oxygen pressure, PTiO_2_; cerebral perfusion pressure, CPP; systolic arterial blood pressure, SABP; tidal volume, Vt; respiratory rate, RR; Plateau pressure, PP; driving pressure, DP; mechanical power, MP; intracranial pressure, ICP; oxygen pressure at half arterial oxygen pressure, p50; optic nerve sheath diameter, ONSD; pulsatility index, PI; Computed Tomography, CT

Oxygen (O_2_) is vital for neuronal survival [1]. Since the brain cannot store O_2_, it needs its constant supply to maintain its main energy source, which is adenosine triphosphate [1]. Two requirements are essential to ensure the availability of O_2_ to the brain (DO_2_): sufficient cerebral blood flow (CBF) and adequate arterial oxygen content (CaO_2_) [1, 2]. Under physiological conditions, the brain utilizes only 33% of the O_2_ received, being able to increase extraction when DO_2_ is compromised in any of its determinants [1, 2]. The achievement of oxygen final metabolism, in the mitochondria, starts from ambient air or a gaseous mixture provided by non-invasive O_2_ supplemental techniques or mechanical ventilator. This requires a good functioning of the respiratory, cardiovascular (including microcirculation) and hematological systems, all regulated by the state of the internal steady [1, 2]. When DO_2_ is inadequate or the mitochondria cannot use the supplied O_2_, ‘‘cerebral tissue hypoxia’’ (CTH) occurs, which constitutes a secondary insult that magnifies the primary brain injury and worsens clinical outcomes, especially in severe traumatic brain injured patients [2]. DO_2_ is the result of CBF x CaO_2_, which does not allow the detection of local tissue or microcirculatory abnormalities that limit the local supply of O_2_ at the tissue level (Fig. 1A and B). CTH is common and prevalent in neurocritical ill patients, and in most cases, it is due to changes in basic physiological parameters [3]. The etiologies of CTH are multiple [4] (Table 1), and can be pathophysiologically approached and investigated following the oxygen route [5] (Fig. 1A and B). However, monitoring of cerebral oxygenation in traumatic brain injured patients is not routinely applied, it has some limitations, and the evidence-based support is not so solid [6, 7]. Even in developed countries, O_2_ tissue pressure (PtiO_2_) monitoring rates do not exceed 19% of centers [8]. A recent study suggests that only 8.6% of centers use routinely PtiO_2_, 1.3% use venous saturation of the jugular bulb (SvjO_2_) and 1.7% near infrared spectroscopy (NIRS) [9]. Determinants of cerebral oxygenation are multifactorial, and a personalized clinical approach depends on the individual pathophysiological causes. To keep in mind in a practical and simple way the physiological variables involved in the transport and utilization of O_2_, and help decreasing the occurrence of episodes of cerebral hypoxia, we have created the mnemonic ''THE MANTLE,'' which can be a useful tool at bedside to remind the factors that protect and optimize cerebral oxygenation in severe traumatic brain injured patients (Fig. 2).

**Table 1:** Causes and types of cerebral hypoxia

	CBF	PtiO_2_	LPR	OEF	Pathophysiology
Ischemic	↓	↓	↑	↑	Inadequate CBF
Low extraction	≅	↓	↑	≅	Low arterial partial pressure of oxygen, PaO_2_ (hypoxemic hypoxia)
					Low hemoglobin concentration (anemic hypoxia)
					Low half-saturation pressure P50 (high- affinity hypoxia)
Shunt	↑	≅	↑	↓	Arteriovenous shunting (microvascular shunt)
Diffusion	≅	≅	↑	↓	Diffusion barrier (intracellular or interstitial edema)
Uncoupling	≅	≅	↑	↓	Mitochondrial dysfunction
Hypermetabolic	↑	↓	↑	↑	Increased demand

CBF: cerebral blood flow; PtiO_2_: brain oxygen pressure; LPR: lactate/pyruvate ratio; OEF: oxygen extraction fraction

### Temperature: “To avoid hyperthermia is fundamental”

Hyperthermia is highly prevalent in neurocritical patients [10–12]. During the initial phase of brain injury, temperature elevation is commonly attributed to the acute phase response, with inflammatory activation and increased sympathetic activity. Direct damage to the hypothalamic thermoregulatory centers can also cause hyperthermia [10–12]. The brain is warmer than the body, and the difference between brain and central temperature may be up to 2ºC [10, 11]. The presence of fever at ICU admission or during the first hours of evolution constitutes a secondary insult that is associated with the severity of the injury, negatively impacting the final outcomes [10, 11]. Hyperthermia exerts its deleterious effects through various mechanisms: increased levels of excitatory amino acids and free oxygen radicals, inhibition of proteolytic enzymes, rupture of the blood–brain barrier and increased ischemic area in vulnerable regions [10, 11]. Hyperthermia can also yield to cerebral hypoxia due to increased metabolism. Therefore, it is desirable to maintain central temperature levels between 36 and 37ºC [10–12].

### Hemoglobin (Hgb): ‘‘To keep and maintain good quality and quantity of transporter is essential’’

Hgb transports more than 95% of the O_2_ in the blood [1, 13]. Physiologically, each unit decrement in Hgb levels reduces O_2_ transport capacity (anemic hypoxia), while oxygen delivery does not increase when Hgb values are higher (> 12 gr/dl) [1, 13, 14]. Transfusions do not ensure the correction of cerebral hypoxia due to multiple variables, including amount of blood transfused, and age of donor [3]. The optimal levels of Hgb remain unknown; however, it seems reasonable to reach and maintain Hgb values between 7 and 9 gr/dl [13]. Blood stored for long periods of time decreases its component of 2,3 diphosphoglycerate, which further increases the affinity of Hgb for O_2_, restricting cell availability [1, 14].

### Electrolytes and acid basic status: ‘‘Physiological balance is the cornerstone’’

Homeostasis of the cellular exterior environment is a key factor to ensure physiology of the transport and cession of O_2_ to the cells. This plays an essential role in avoiding shifts in the Hgb dissociation curve [1, 2, 10]. Both the increase in temperature and carbon dioxide (CO_2_) and tissue acidosis, product of cellular metabolism, facilitate the transfer of O_2_ to the tissues by shifting the O_2_/Hgb dissociation curve to the right. In contrast, hypothermia, hypocapnia, and alkalosis increase the affinity of Hgb for O_2_ (shift to the left), which makes it more difficult to transfer the necessary O_2_ to the cell [1]. Acidosis, hypercapnia, and hyperthermia dilate cerebral resistance blood vessels, increasing cerebral blood volume and intracranial pressure, while hypocapnia, by causing vasoconstriction, facilitates cerebral ischemia [1]. In order to ensure that Hgb dissociation curve remains within functional ranges (p50 = 26–28 mmHg), to reduce the risk of cerebral ischemia and intracranial hypertension, the following goals should be achieved: a) pH: 7.35–7.45; b) normocapnia; c) central temperature (T°): 36–37.5 °C [1, 10]. On the other hand, to minimize or treat cerebral edema, it is crucial to maintain a slight hyperosmolar state (serum Na^+^ 140—150 mEq/L) and to avoid hypotonic fluids [15].

### Metabolism: ‘‘If metabolism is accelerated, O_2_ demands increase’’

Brain metabolism is the main determinant of the rate of cerebral O_2_ consumption. In some cases of hypoxia, O_2_ demands exceed supply. For this reason, all those situations that increase the neuronal demand for O_2_, such as inadequate level of sedation and analgesia (pain, agitation), seizures, fever, sepsis, and paroxysmal sympathetic hyperactivity syndrome, should be investigated and quickly corrected [1, 13, 16]. The cerebral oxygenation goals to be achieved depend on the available resources and the technique employed. Oxygen pressure of the brain parenchyma locally reflects the balance between the supply and consumption of O_2_ and should be maintained at values above 18 mmHg. The venous oxygen saturation obtained from the jugular bulb (SvjO_2_), globally represents the O_2_ that returns to the general circulation after being consumed by brain cells and should be maintained at values > 55%. Both variables depend on adequate CBF, which in turn requires appropriate CPP. When advanced and specialized technology is available, such as microdialysis or a specific software for the continuous evaluation of the autoregulatory phenomenon, it is recommended to maintain the lactate/pyruvate ratio < 25 and pressure reactivity index (Prx) or oxygen reactivity index (Orx) < 0.2. Orx and Prx are the correlation coefficients between CPP and PtiO_2_ and ICP, respectively. Both parameters are related to cerebral oxygenation, as high ICP reduces oxygen tolerance.

### Arterial blood pressure: ‘‘Arterial hypotension is apocalyptic for injured brain’’

One of the main determinants of CBF is CPP, which is the result of mean arterial blood pressure (MABP) minus intracranial pressure (ICP), and depends on the diameter of small cerebral blood vessels (50–150 microns) [13]. These parameters interact giving rise to the cerebral autoregulation curve, an intrinsic phenomenon of resistance in blood vessels that allows, by changing their diameter, to maintain constant CBF [13]. This property is not unlimited, and when autoregulation is impaired, CBF may passively follow the CPP above or below the limits. For years, it has been considered that CBF does not change despite fluctuations in CPP in the range of 50 to 150 mmHg [17]. Recently, physiological studies have challenged this assertion, showing that the phenomenon of cerebral autoregulation is more ''passive'' and the ''plateau'' phase of the autoregulation curve is considerably narrower in brain injured patients [18]. CBF can vary and even become pressure dependent even in physiological situations where blood pressure (BP) varies abruptly such as exercise [18]. Cerebral autoregulation (CAR) is critical to maintain proper brain perfusion and oxygenation. PtiO_2_ is a surrogate of CBF [19, 20]. CAR can be easily monitored by transcranial Doppler [21] or by an invasive way through MABP manipulation [22] or the utilization of specific software that established the correlation between brain tissue oxygenation and CPP [19, 20]. Orx is an index that evaluates CAR through the relationship between CPP and PtiO_2_. Orx may vary between − 1 and + 1. When PtiO_2_ passively follows CPP, autoregulation is compromised, so a positive correlation exists [19, 20]. When autoregulation is intact, PtiO_2_ is not affected by changes in CPP so there is inverse correlation between both parameters and Orx [19, 20].In turn, MABP depends on different hemodynamic variables such as systemic vascular resistance and cardiac output. In traumatic brain injury, arterial hypotension is one of the factors with the greatest negative impact on the final outcome and can contribute to the development of ischemic hypoxia, and therefore must be urgently prevented and corrected [5, 13].

Recommended blood pressure targets include systolic blood pressure > 100–110 mmHg; normal volemia, diuresis > 30 ml/h, preserved peripheral perfusion, central venous pressure: 6–10 cmH_2_O [5, 7, 13]. Acceptable CPP levels do not ensure normal brain oxygenation, since there is evidence that brain tissue hypoxia can occur even with normal MABP and ICP values [5]. Furthermore, the concept of personalization of treatment is gaining interest, based on the concept that clinicians should not only consider a common pathophysiological pathway independently from specific brain damage but should adapt the therapeutic management to specific needs [23–26]. For example, one parameter to be considered could be the volume of the contusion [19]. In the presence of a small contusion, the blood brain barrier (BBB) is closed in most of the brain. In this case: 1) the osmolarity is the main driving force for edema formation; 2) autoregulation is efficient (increasing pressure decreases cerebral blood volume); so, 3) the first line treatment may include cerebral fluid drainage, increase in the CPP and osmotherapy [26]. When the contusion volume is greater, the BBB is at least partially open, so: 1) higher osmolarity and pressure may worsen edema; 2) vasogenic edema should be prevented in the contusion area; 3) the first line treatment includes cerebral fluid drainage, deep sedation and perhaps hypothermia [26].

### Nutrition and glucose: ‘‘Glucose, essential fuel for the damaged brain’’

Glucose is an essential nutrient and energy substrate to maintain mitochondrial functionality [27]. The injured brain increases its avidity for glucose, and as there is no storage of glucose, no more than 2 min of glucose deprivation are necessary to deplete the scarce cerebral reserves [27]. The consequences of the little availability of glucose to the brain are the main reasons for metabolism compromise. Glycemia levels < 110 mg/dl may cause non-ischemic metabolic crises [28]. In contrast, hyperglycemia > 180 mg/dl causes neurotoxic cascades (inflammation, micro thrombosis, edema) and disturbs the homeostasis of the internal environment (hyperosmolarity, dehydration), compromising the immune status, among other alterations [27]. In addition, neuroglycopenia can contribute to mitochondrial dysfunction (uncoupling hypoxia) [28].

### Target of oxygenation: ‘‘Both extremes of systemic oxygenation are deleterious’’

Systemic oxygenation strictly depends on lung function, and the variables that determine gas exchange, especially the ventilation/perfusion ratio and its extremes (dead space and shunt) must be within physiological limits [13]. The increase in dead space causes a decrease in alveolar ventilation, which causes CO_2_ retention and hypoxemia. On the other hand, the increase in the shunt fraction generates hypoxemia because mixed venous blood perfuses large non-ventilated areas, not allowing arterial blood to become enriched in O_2_. Markers of this type of hypoxemic hypoxia are the decrease in arterial oxygen pressure (PaO_2_) and arterial oxygen saturation (SaO_2_), bearing in mind that PaO_2_ represents dissolved O_2_, that affects only 3–4% of the total of oxygen transport capacity [1].

In this context, a common and rational practice would be to increase the fraction of inspired O_2_ (FiO_2_); however, this measure does not solve the underlying problem without an exhaustive analysis of the situation, since even with supranormal levels of PaO_2_ (normobaric hyperoxia) hidden cerebral hypoxia can still occur; on the other side, recent evidence suggested that even hyperoxia can be harmful [29].

If these variables are compromised, measures must be taken to achieve PaO_2_ 80–120 mmHg, and SaO_2_ > 95% [30].

### Lung protective ventilation: ‘‘Protecting the lungs protects the brain’’

The concept of lung protective ventilation is challenging in brain injured patients. In fact, the combination of low tidal volume (to keep low plateau pressure and driving pressure) with high intrathoracic pressures and reduced venous outflow induced by positive end expiratory pressure (PEEP) might favor an increase in carbon dioxide value. For these reasons, traditionally, this population of patients has been excluded from the major trials investigating protective strategies in the general ICU population, and no strong evidence is available on this topic [30, 31].

However, over the last years, the concept of lung protective ventilation is gaining interest even in brain injured patients, as it can reduce pulmonary complications and therefore be associated with improved outcomes [30, 31].

Optimizing mechanical ventilator strategies means optimizing lung function and systemic and cerebral oxygenation, but at the same time reducing the risk of ischemic hypoxia secondary to vasoconstriction (hypocapnia) and intracranial hypertension for vasodilatation (hypercapnia) [3, 5, 7].

According to available evidence, it seems prudent to start lung protective ventilation with a controlled mode, tidal volumes between 6 and 8 ml/ kg, minimum respiratory rates to ensure levels of PaCO_2_ between 35 and 45 mmHg, and FiO_2_ and PEEP necessary to achieve systemic oxygenation targets as we mentioned above [30, 31]. To prevent mechanical ventilation induced lung injury (barotrauma, biotrauma, volutrauma) plateau pressure should be kept < 2 cmH_2_O, driving pressure < 13 cm H_2_O [30, 31] and mechanical power below 17 J/min [32]. It is recommended not to use routinely hyperventilation and to maintain PaCO_2_ levels between 35 and 45 mmHg [7]. Lower targets can be used as strategies to control intracranial hypertension [22]. In life threatening situations, such as herniation syndromes, plateau waves type A or intracranial hypertension secondary to hyperemia, moderate and controlled hyperventilation can be used [33, 34].

### Edema and ICP control: ‘‘Brain swollen, brain on the ledge’’

Cerebral edema contributes to the development of cerebral tissue hypoxia through two mechanisms. On one hand, it can cause ischemic hypoxia by increased ICP with consequent decrease in CPP; on the other hand, it contributes to the development of hypoxia by reducing diffusion of O_2_ to the cells [35, 36], Fig. 2. Achievement of appropriate levels of sodium is essential to minimize cerebral edema [15]. Also, the application of established intracranial hypertension management protocol is recommended to treat intracranial hypertension [22, 37–40]. The recommended main targets to be achieved should be the following: a) ICP < 22 mmHg; b) CPP: 55–70 mmHg; c) optic nerve sheath diameter (ONSD) < 5.8 mm; d) pulsatility index (PI) < 1.2; and e) Cerebral CT scan without edema signs.

## Conclusion

CTH is not uncommon in severe traumatic brain injury and independently predicts poor outcomes. Knowledge of the physiology and kinetics of O_2_ and of the various causes of hypoxia, together with clinical reasoning and personalized treatment can help to minimize the incidence of CTH and its direct and dangerous consequences even without advanced and specific neuromonitoring.

## Data Availability

Not applicable.

## Abbreviations

O_2_: Oxygen
DO_2_: Oxygen availability
CBF: Cerebral blood flow
CTH: Cerebral tissue hypoxia
PtiO_2_: Brain oxygen tissue pressure
SvjO_2_: Jugular bulb venous oxygen saturation
NIRS: Near infrared spectroscopy
Hgb: Hemoglobin
ºC: Centigrade degrees
ICP: Intracranial pressure
CPP: Cerebral perfusion pressure
MABP: Mean arterial blood pressure
BP: Blood pressure
CAR: Cerebral autoregulation
TBI: Traumatic brain injury
paO_2_: Arterial partial pressure of oxygen
SaO_2_: Arterial oxygen saturation
FiO_2_: Inspired oxygen fraction
p50: Oxygen pressure at half arterial oxygen saturation
paCO_2_: Arterial carbon dioxide pressure
PEEP: Positive end expiratory pressure
OSND: Optic sheath nerve diameter
PI: Pulsatility index
CT: Computed tomography
